# Craniofacial and olfactory sensory changes after long-term unilateral nasal obstruction—an animal study using *MMP-3*-LUC transgenic rats

**DOI:** 10.1038/s41598-024-51544-3

**Published:** 2024-01-31

**Authors:** Li-Fang Hsu, Nutthakarn Ratanasereeprasert, Shih-Kai Wang, Jung-Tsu Chen, Yi-Jane Chen, Te-Huei Yeh, Hsiang-Hsuan Sung, Chung-Chen Jane Yao

**Affiliations:** 1 Department of Dentistry, National Taiwan University Hospital, Hsin-Chu Branch, Hsin-Chu, Taiwan; 2 Graduate Institute of Clinical Dentistry, School of Dentistry, National Taiwan University, No. 1, Chang-Te Street, Taipei, 10048 Taiwan; 3 Department of Dentistry, National Taiwan University School of Dentistry, Taipei, Taiwan; 4 Division of Orthodontics and Dentofacial Orthopedics, Dental Department, National Taiwan University Hospital, Taipei, Taiwan; 5 Department of Otolaryngology, National Taiwan University Hospital, College of Medicine, National Taiwan University, Taipei, Taiwan; 6 National Laboratory Animal Center, National Applied Research Laboratories, Taipei, Taiwan

**Keywords:** Bone, Bone development, Bone remodelling

## Abstract

Nasal obstruction exerts considerable physiological effects on the respiratory system and craniofacial morphology during the developmental stage. This study used *MMP-3*-LUC transgenic rats for in vivo tracking of long-term expression in the rat nasal region after unilateral nasal obstruction. Skeletal changes of the craniofacial, nasal, and sinus regions were measured through micro-computed tomography examination and analysis with 3D image processing and calculation. Matrix metalloproteinase-3 and olfactory marker protein expression were also investigated through immunohistochemistry (IHC). Unilateral nasal obstruction significantly reduced the MMP-3 signal in the nasal region of *MMP-3*-LUC transgenic rats, which was mainly expressed in the respiratory epithelium. Long-term obstruction also caused morphological changes of the craniofacial hard tissue, such as nasal septal deviation, longer inter-jaw distance, and increased maxillary molar dental height. It also caused compensatory growth in olfactory nerve bundles and the olfactory epithelium, as confirmed by IHC. In our study, long-term unilateral nasal obstruction caused nasal septal deviation toward the unobstructed side, hyper divergent facial development including longer molar dental height, and reduced MMP-3 production. However, further investigation is necessary to explore the mechanism in depth.

## Introduction

Nasal obstruction, whether resulting from chronic rhinosinusitis, infection, or other organic problems such as severe septal deviation, interferes with craniofacial skeletal development, alters olfactory function and taste, and leads to abnormal compensatory tongue posture^1–5^. The volume of the maxillary sinus has also been found to be affected by the pathophysiological state of the sinus epithelium; previous studies have reported that patients with chronic rhinosinusitis tend to have a smaller maxillary sinus volume^6,7^. However, the mechanism by which the bony sinus wall is remodeled under healthy or diseased epithelium remains unknown.

The process of bone resorption includes the elimination of organic and inorganic substances. Degradation of the extracellular organic matrix relies on proteinases such as lysosomal cysteine proteinase, serine proteinases, and matrix metalloproteinases (MMPs). MMPs are a group of calcium-dependent zinc-containing endopeptidases that can hydrolyze extracellular matrix proteins and basement membranes such as collagen, elastin, glycoproteins, and proteoglycans. Among them, MMP-3 is typically involved in tissues under pathological and inflammatory changes like tumors or arthritis joints^8,9^. Nonetheless, it’s physiological expression was also reported in salivary glands, retina, and brain^10–12^. It was also reported to participate in bone remodeling in reaction to mechanical force, such as compressive and tensile forces, as an early effector protein. Previous research has confirmed up-regulation of the MMP-3 gene under compressive force in human osteoblast cell lines, increased MMP-3 protein expression in human alveolar bone, and positive green fluorescent protein (GFP) signaling in the periodontal ligament of MMP-3-GFP mice that underwent orthodontic treatment^13,14^. The respiratory epithelium is the first direct mechanosensory organ of respiratory airflow, and its reaction to the blockage of airflow may affect long-term nasal and sinus formation. MMP-3 has been reported to account for cell proliferation in the naso-epithelial cell line^15^, and it was also clearly expressed around nose region in our *MMP-3*-LUC transgenic rats (Supplementary Fig. S1). Thus, our hypothesis is that MMP-3 participates in nasal formation in response to the presence or blockage of airflow.

The present study used *MMP-3*-LUC transgenic rats for long-term tracking of MMP-3 expression in the rat nasal region after unilateral nasal obstruction. Craniofacial, nasal, and sinus skeletal changes as well as olfactory sensory input changes were investigated through micro-computed tomography (CT) and immunohistochemistry (IHC).

## Results

### Evaluation of validity of nasal obstruction

To determine the effectiveness of the silicone method used in the experiment, micro-CT was used to confirm intranasal presence of the material each month. A material stability test was also performed to confirm the stability after soaking under saline without shrinking or expansion of the volume. The material was very stable in the nasal compartment throughout the experiment with very little volumetric changes observed (Table 1).

**Table 1:** Volume changes of the 5 silicone blocks in each group after a 5-month period stored under dry and wet conditions (control group: dry conditions in an incubator with 5% CO_2_ and humidified conditions; experimental group: soaked in normal saline in an incubator with 5% CO_2_ and humidified conditions).

	Control	Control_5M	Exp	Exp_5M
Average (mm^3^)	833.343	851.586	816.508	823.881
Standard deviation (mm^3^)	19.300	14.321	21.192	22.666
% of volume change	2%		1%	

Cone-beam CT was used to acquire the block dimensions and the images were then analyzed using Amira software.

### Animal experiment and in vivo *MMP-3*-LUC signals

**Figure 1:**
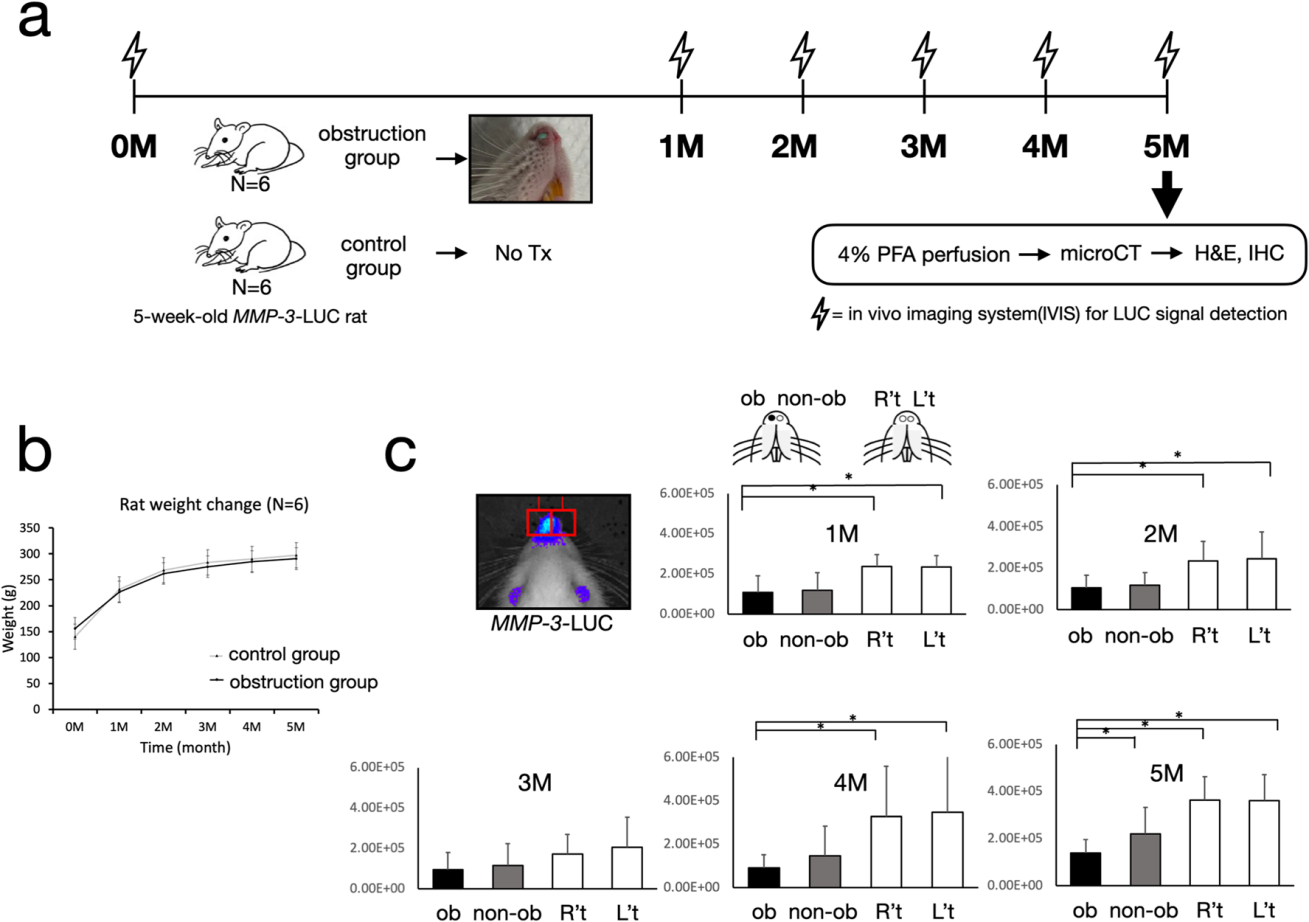
Experiment flow of unilateral nasal obstruction using an MMP-3-luciferase transgenic rat model (**a**) and in vivo imaging results. The monthly recorded weight showed no significant differences in the two groups (**b**). In vivo luciferase detection in the nasal region in the experiment period showed a significant decrease in the experimental (nasal obstruction) group (**c**). *p < 0.05.

All animals maintained normal weight gain throughout the experiment. The experimental group had a lower weight than the control group after 5 months, but without statistical significance. The *MMP-3*-LUC signal in the experimental group showed an obvious reduction on both the obstruction side and non-obstruction side of the nose compared to the control group. The LUC signal was significantly reduced over the obstruction side of the nose at 1, 2, 4, and 5 months (*p* < 0.05) (Fig. 1). In the experimental group, the signal was also reduced over the obstruction side compared with the non-obstruction side at 5 months (*p* < 0.05). The signal of either side of the control group was similar, without significant differences.

### Investigation of long-term effects of nasal obstruction on nasal and craniofacial development

**Figure 2:**
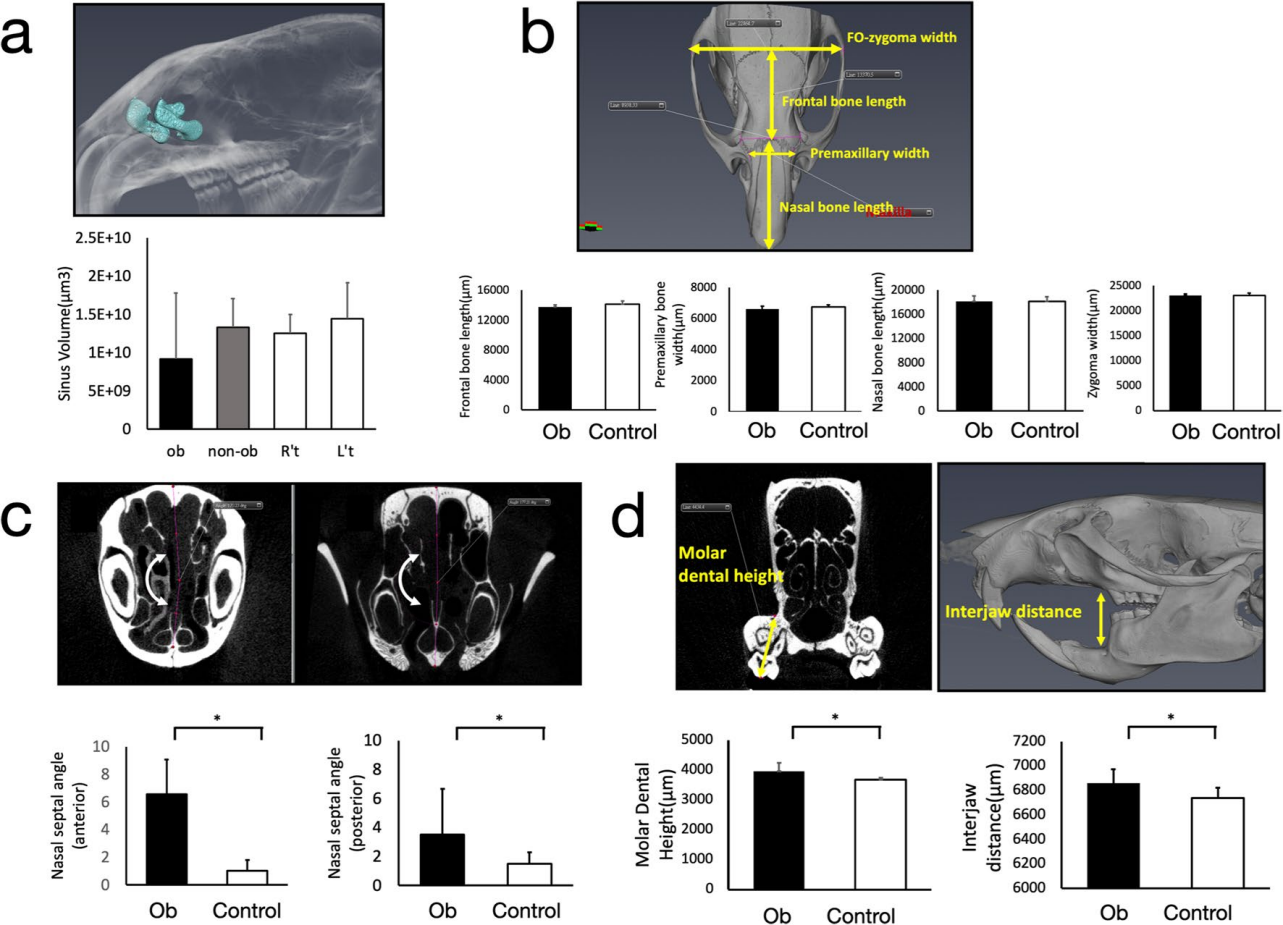
Micro-CT analysis of maxillary sinus volume, craniofacial skeletal dimension, nasal septal deviation angle, dental height, and interjaw distance measurements. (**a**) Maxillary sinus volume calculation. The black bar and shaded bar indicate measurements in the experimental group, whereas the white bars indicate left and right side measurements in the control group. (**b**) Craniofacial measurements. (**c**) Anterior and posterior nasal septal deviation angle. (**d**) Maxillary molar dental height and interjaw distance measurements. *p < 0.05.

To investigate the effect of unilateral nasal obstruction on craniofacial, nasal, and sinus development, micro-CT scanning was performed for each rat at the end of the experiment. Using Amira calculation software (Version 2022.1, Thermo Fisher Scientific, Waltham, MA, USA), the volume of each side of the maxillary sinus was calculated and compared. Although a tendency was observed in the experimental group for both sides of the nose to have a slightly lower maxillary sinus volume compared to the control group, the difference was non-significant. The widths and lengths of the cranium also exhibited no significant differences between the experimental and control groups (Fig. 2a,b).

Measurement of nasal septum deviation revealed that the experimental group had a significantly more deviated septum compared to the control group (anterior angle: Ob group: 6.56 ± 2.51 degrees vs. control group 1.13 ± 0.80 degrees; posterior angle: Ob group 3.51 ± 3.17 degrees vs. control group 1.47 ± 0.82 degrees), which was also confirmed by comparing serial micro-CT images from each month (Fig. 2c, Supplementary Fig. S2).

Finally, in terms of the vertical development, the molar dental height and inter-jaw distance were both significantly increased in the groups with obstructions on either side of the nose compared to the control group (Fig. 2d).

### Inhibition of MMP-3 expression of the nasal respiratory epithelium and increased OMP expression under the olfactory epithelium

**Figure 3:**
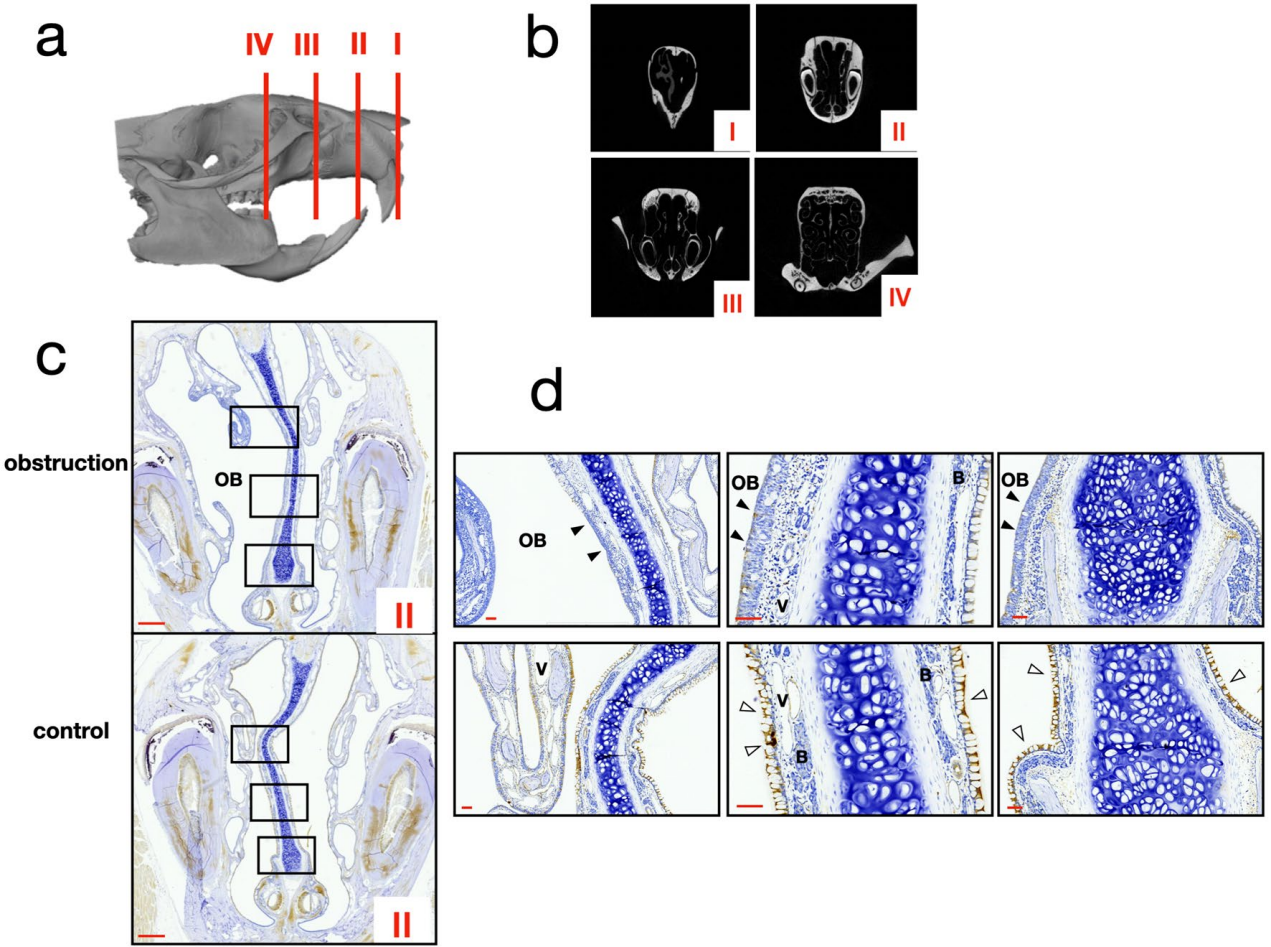
(**a**, **b**) Frontal sections of the nasomaxillary complex defined in this study from the rostral to caudal ends: Section I: from the nostrils to a complete bony nasal wall without a vomeral nasal organ and incisor roots. Section II: the vomeral nasal organ without a zygomatic process. Section III: from the start of the zygomatic process to the position of the maxillary 1st molar. Section IV: nasal turbinates and maxillary molars. (**c**) IHC staining of MMP-3 at Section II: comparison of the obstruction and control groups. Scale bar indicates 1 mm. (**d**) Upper row: images from the obstruction group with higher magnification, showing epithelial transition and a decrease in MMP-3 staining, particularly over the obstruction side. Submucosal vessels on the obstruction side also decreased substantially. Lower row: obvious MMP-3 staining on the respiratory epithelium over both left and right sides ini the control group. (V, vessels; B, Bowman’s gland; black arrowhead, epithelial transition; white arrowhead, positive MMP-3 staining) Scale bar indicates 100 µm.

Histological examination revealed that the nasal epithelium from the obstruction site at different section levels were distinct from the non-obstruction site. At rostral levels (Level II) of the respiratory epithelium exhibited less vascularization and less Bowman’s gland in the lamina propria on the obstruction side. Furthermore, the respiratory epithelium changed toward stratified epithelium without goblet cells, and an absent basement membrane layer on the obstruction side. IHC revealed decreased MMP-3 staining on both sides of the respiratory epithelium in the obstruction group compared to the control group, which echoed the results of IVIS signal changes (Fig. 3).

**Figure 4:**
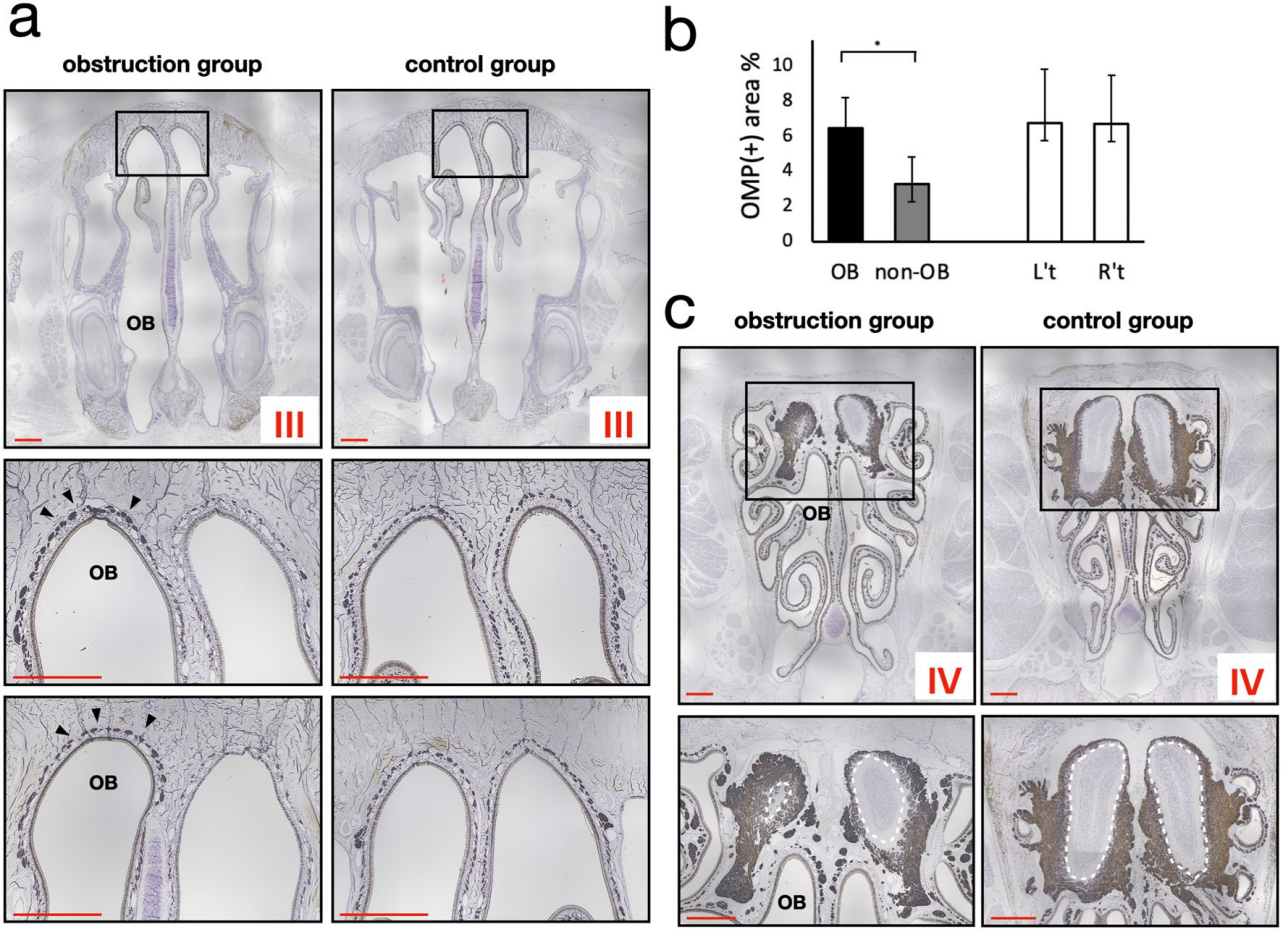
IHC of OMP staining at Section III and Section IV. (**a**) Obstruction group showing obvious enhancement of OMP expression on the obstruction side (the side marked **OB**). In the control rats, the distribution of OMP was even on both sides. (**b**) Quantification of the OMP-positive area in two groups. (**c**) OMP staining at Section IV, showing obviously reduced olfactory bulb size in the obstruction group compared to the control group. (Black arrowhead, positive OMP staining; white dash line depicts the outline of olfactory bulb inside the glomerular layer.) *p < 0.05. Scale bar indicates 1 mm.

More caudal levels (Level III) exhibited keratinization in the superficial layer of the olfactory epithelium with densely populated neurons accumulated at the base. Clusters of thick neuronal bundles occupied more than half of the submucosal spaces in addition to significant reduction of submucosal Bowman’s glands. IHC showed a strong OMP-positive signal at those neuronal bundles compared to very little expression on the contralateral side. In the control group, both sides showed even distribution of OMP-positive neuronal fibers (Fig. 4a,b). At level IV, olfactory bulb size was reduced especially in the obstruction side of the obstruction group. The OMP-positive glomeruli layer had compensatory thickening compared to the patent side of the obstruction group (Fig. 4c).

## Discussion

The silicone material showed high stability in the 5-month stability test (Table 1).

Many protocols have been developed for reversible or irreversible rodent nasal obstruction: cauterization of nostrils^3,4^, injection of synthetic resin/ sponge/ silicone material^2,16,17^, or direct suturing to close the nostril^5^. Although all methods can induce successful nasal obstruction, silicone materials are very stable after setting, can be inserted without physical trauma, and have the fewest adverse effects.

Accordingly, a normal weight growth curve was observed in the experimental group as well as the control group(Fig. 1b), which echoed previous research using a similar method^2^. It also reflects the condition in humans because patients with nasal obstruction or allergic rhinitis do not exhibit a tendency toward lower body weight^18–20^. The experiment duration of from 5 weeks to 5 months were chosen because the craniofacial growth of rat peaks between week 5 to week 7, and reached puberty at around 3 months. We extended further to see if compensatory changes occurred.

In the present study, a slightly decreased sinus volume was observed in the control group but without significant differences, due to high variability (Fig. 2a). However, occupied sinus volume was found increased in the obstruction group compared to the control group, suggesting that prolonged nasal obstruction may result in intra-sinus soft tissue changes or pathological changes occupying the maxillary sinus.

A decrease in maxillary sinus volume has been associated with chronic pathological conditions such as rhinosinusitis^6,7^.

A relationship between oral breathing and craniofacial changes has been reported in many species. Knowing that rodents is obligate nasal breather, it is also true that rodents can become mouth-breathing under stress, illness, or respiratory disease conditions, with an apparently higher death rate^21,22^. With that, many studies conducted in rodents had accomplished the task of not only unilateral nasal obstruction, but also bilateral nasal obstruction with a successful outcome^4,23–26^.

In brief, the findings were mainly smaller dimensions of the craniofacial skeleton^1,17^. Vertical growth of the maxillary complex has varied in past research: studies using Macaca monkeys have shown increased maxillary height under the effect of nasal obstruction^27,28^, whereas other studies on rats have shown significantly reduced nasomaxillary height caused by obstruction. In our study, the inter-jaw distance and maxillary molar dental height significantly increased in the unilateral nasal obstruction group, both of which might be due to increased oral breathing proportion. The other craniofacial measurements showed no differences in height and width, meaning that in the present study, the effect of oral breathing was more confined to alveolar bone and condylar position changes rather than the overall skull development. This is closer to the observations that we saw in human: no differences are apparent in the skull dimensions or body weight/height of humans with prolonged oral breathing. However, a more dolichofacial growth pattern can gradually develop as a result of oral breathing and longer dental heights.

In the present study, the nasal septal deviation angle increased significantly both in anterior and posterior parts in the obstruction group compared to the control group (Fig. 2c). The forming process was confirmed by monthly micro-CT images (Supplementary Fig. S2). Septal deviation is mainly attributable to two factors: injury during birth and trauma. It has been regarded as a traumatic injury rather than a developmental defect^29,30^. The current study revealed another possible cause of septal deviation during developmental stage. The need to regain airway patency on the obstruction side is assumed to be the main reason for septal deviation, which might resemble the clinical condition in which pediatric patients with prolonged nasal obstruction can eventually develop a deviated septum. To our knowledge, this is the first study to report the formation process of septal deviation.

The *MMP-3*-LUC transgenic rat model confirmed MMP-3 mRNA expression under normal physiological conditions in the rat nose region and IHC showed that the expression was located in the respiratory epithelium. This expression was suppressed after nasal obstruction, even on the non-obstruction side of the experimental group, meaning that the deprivation of airflow results in the down-regulation of MMP-3. IHC confirmed that both the obstruction and non-obstruction sides of the experimental group exhibited reduced MMP-3 staining. MMP-3 has been found to participate in many physiological processes, such as tissue remodeling, neuronal growth, and cell differentiation^15,31–34^. It was also reported to be involved in epithelial-mesenchymal transition in the respiratory epithelium and mammary epithelial cells^35,36^, and in cell proliferation in the nasal epithelial cell line^15^. In our flow experiment of the primary naso-epithelial cells, the cells responsible for physiological MMP-3 expression were found in fibroblast-like cells but not epithelial-like cells with tight junctions. One assumption is that MMP-3 participates in the regulation of the natural degradation and renewal process of the respiratory epithelium, as well as the development of the airway space. This process may be controlled via a mechanical force-mediated pathway, or immune-mediated pathway. Mechanosensory feedback from epithelial cells induces MMP-3 expression and increases cell proliferation, thus expanding the airway surface. The activation of immune responses can also trigger MMP-3 release, leading to cascades dissolving the extracellular matrix and the leakage of tight junctions, allowing immune cells to travel. However, further research is necessary to elucidate the signal transduction process.

Olfactory marker protein is a small cytoplasmic protein expressed in nasal olfactory sensory neurons and other nasal chemosensory neurons^37,38^. Although it is commonly recognized as an olfactory receptor marker, its function is still not clearly understood. Previous research has established its role in early olfactory signal transduction, as well as buffering of cAMP to maintain resilience of olfactory receptor neurons^37–39^. Our results revealed significantly enhanced immunoreactivity of OMP on the obstruction side of the olfactory epithelium, and underlying structures compared to the contralateral side. Increased neuronal bundles are responsible for OMP staining in the lamina propria, whereas the nuclei of olfactory sensory neurons are located at the base of the cell with denser cilia distribution. This finding shows a compensation of the neuronal sensory mechanism, suggesting that olfactory function and development might not be directly related to the presence of airflow, and diminished airflow can somehow further stimulate neuronal growth. This result also explains the preservation of olfactory function through enhanced sensory inputs in previous studies^40–42^. The compensatory mechanism was established in previous research by Coppola et al.; however, the result differs from evidence that sino-nasal diseases and pathogens cause degeneration of the olfactory epithelium^43^, primarily because of damage related to pathological conditions.

The results of this study revealed that long-term unilateral nasal obstruction from prepubertal stage can cause septal deviation, longer inter-jaw distance, and increased maxillary dental height in rodents. The growth of olfactory nerve and olfactory bulb were also affected. Nonetheless, the current study still has its limitations, such as lack of monitoring of nasal airflow and a low sample size. Future aims should be focused on the addition of obstruction-reversal group to see if changes in MMP-3, craniofacial skeleton, and olfactory growth can be reversed.

## Conclusion

In LUC-MMP-3 transgenic rats, unilateral nasal obstruction can significantly reduce the MMP-3 signal, which is mainly expressed in the respiratory epithelium. This long-term obstruction also caused morphological changes in the craniofacial hard tissue, such as nasal septal deviation, longer interjaw distance, and increased maxillary molar dental height. Unilateral nasal obstruction also caused compensatory growth in olfactory nerve bundles and the olfactory epithelium.

### Limitations

This *MMP-3*-LUC transgenic rat model showed physiological expression of this protease in the nose region, requiring further research to elucidate the role of MMP-3 in the upper respiratory tract. Nevertheless, the possibility of reversing organic changes if the deprivation of airflow is recovered also demands further research.

## Methods

### Establishing a model of transgenic rats carrying the MMP-3 human 3.2kb promoter–LUC reporter

Luciferase and GFP bicistronically expressed as an individual protein mediated by a 2A element were first inserted into the rat MMP-3 gene using a Red/ET recombination kit (Gene Bridges, Heidelberg, Germany). This gene in a bacterial artificial chromosome (BAC clone RP230-520D4, obtained from BAC/PAC Resources Center, Oakland Children's Hospital, Oakland, CA, USA) was linearized using Nru I. The resulting 79-kb transgene fragment contained a 46-kb upstream sequence, 23 kb of the MMP-3-Luciferase-2A-emGFP gene, and a 10-kb 3’-sequence. This fragment was purified using pulsed field electrophoresis and gel elution before subsequent pronuclear injection for the generation of transgenic rats.

Genomic insertion was confirmed using genotyping polymerase chain reaction with forward primer GCACCATCTTCTTCAAGGACGAC and reverse primer AACTCCAGCAGGACCATGTGATCG for emGFP at an annealing temperature of 62 °C, resulting in a 380-bp product.

Five lines were identified as having been successfully generated by the Genetic Engineered Murine Model Service, and two lines were characterized and deposited in the Roden Model Resource Center. Transgenic rats were provided, bred and held at by National Laboratory Animal Center (NLAC, NARLabs, Taiwan), and the Animal Center at National Taiwan University College of Medicine. All animal procedures were reviewed and approved by the Institutional Animal Care and Use Committee of National Taiwan University College of Medicine (protocol numbers 20120443, 20130410, 20140495, 20150120, 20160493, and 20180418). All methods were performed in accordance with the relevant guidelines and regulations, including ARRIVE guidelines.

### Animals and the in vivo imaging system for detecting *MMP-3*-LUC expression

Nasal obstruction of the right nostril in *MMP-3*-LUC transgenic rats was performed by injecting 0.1 mL of silicone material (Panasil initial contact light, Kettenbach GmbH & Co. KG, Eschenberg, Germany) into the nostril in the experimental group (n = 6) from 5 weeks of age to 5 months. The control group (n = 6) received no treatment except anesthesia at the same time point as the experimental group. Each month, in vivo luciferase expression was detected using an injection of luciferin (150 mg of luciferin/kg) 20 min prior to scanning with an in vivo imaging system (IVIS). At the experiment end-point, all animals were subjected to deep anesthesia via intraperitoneal injection of a Xylazine and Zoletil™ mixture (Xylazine: Zoletil™ = 1:4) and then sacrificed using the 4% paraformaldehyde perfusion method. Micro-CT of the head of each rat was performed to investigate the craniofacial, nasal, and sinus skeletal developmental changes. Paraffin-embedded tissue sections were then used for histology and IHC.

### Micro-CT scanning and analysis using Amira

At the termination of the experiment, rats were perfused and the heads were soaked in 4% paraformaldehyde for one week before scanning. The scanning was performed on a Skyscan 1276 scanner at an 18-μm pixel resolution, 85 kV, 200 µA, and with a 1-mm AI filter (Skyscan, Kontich, Belgium) and analyzed using Amira software. Linear and angular measurements were directly obtained from 3D reconstruction images and each slice after landmark-defined alignment of the orientation of each rat head. The region of interest was calculated using a segmentation module during sinus volume calculation.

### IHC staining for MMP-3, LUC, and olfactory marker protein

After micro-CT scanning, tissues were decalcified, dehydrated, and embedded in paraffin blocks. Coronal sections (4 µm) were obtained from the nose tip to the caudal end of the maxilla. Hematoxylin and eosin staining was used to identify morphological structures. IHC staining was performed as follows: slides were baked for 30 min, then rehydrated and further heated in sodium citrate buffer (pH = 6) for antigen retrieval. Endogenous peroxidase activity was blocked with 3% H_2_O_2_ for 15 min followed by 30-min incubation in 1% bovine serum albumin to block non-specific binding.

We used the following primary antibodies: anti-olfactory marker protein (1:2000 anti-OMP, ab183947, Abcam, Cambridge, UK), anti-MMP-3 (1:100 ab53015, Abcam), and anti-luciferase (1:2000 Anti-LUC, ab21176, Abcam). The sections were incubated with primary antibodies overnight and washed with tris-buffered saline ((TBS) + 0.05% Tween 20; TBST). Anti-rabbit secondary Fab’ fragments labeled with polymer were added for 30 min and then washed with TBST. We used substrate 3, -3’ diaminobenzidine (DAKO, Glostrup, Denmark) for staining, followed by counter-staining with hematoxylin.

### Statistical analysis

Student’s *t*-test was used to identify statistical significance based on an alpha level = 0.05 and power of 0.8. The error bars in the figures represent standard deviation of the mean. All statistical tests were performed using the Statistical Package for the Social Sciences (IBM, Armonk, NY, USA) and *p*-values < 0.05 were defined as statistically significant.

### Supplementary Information

Supplementary Figure 1.

Supplementary Figure 2.

Supplementary Legends.

## Data Availability

The datasets used and/or analyzed during the current study are available from the corresponding author on reasonable request.
