# Supplementary figures and images for: Craniofacial and olfactory sensory changes after long-term unilateral nasal obstruction—an animal study using MMP-3-LUC transgenic rats
Source: Sci Rep. 2024 Jan 31;14:2616. doi: 10.1038/s41598-024-51544-3 (PMC10830476; doi:10.1038/s41598-024-51544-3)

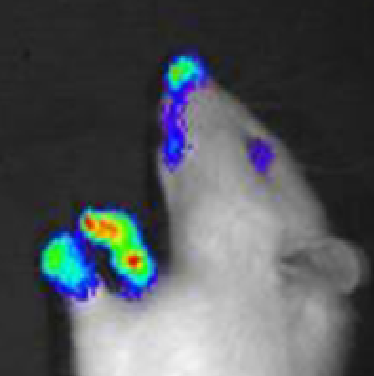

Supplement: Supplementary file 1 — Supplementary Figure 1. [file 41598_2024_51544_MOESM1_ESM.png]

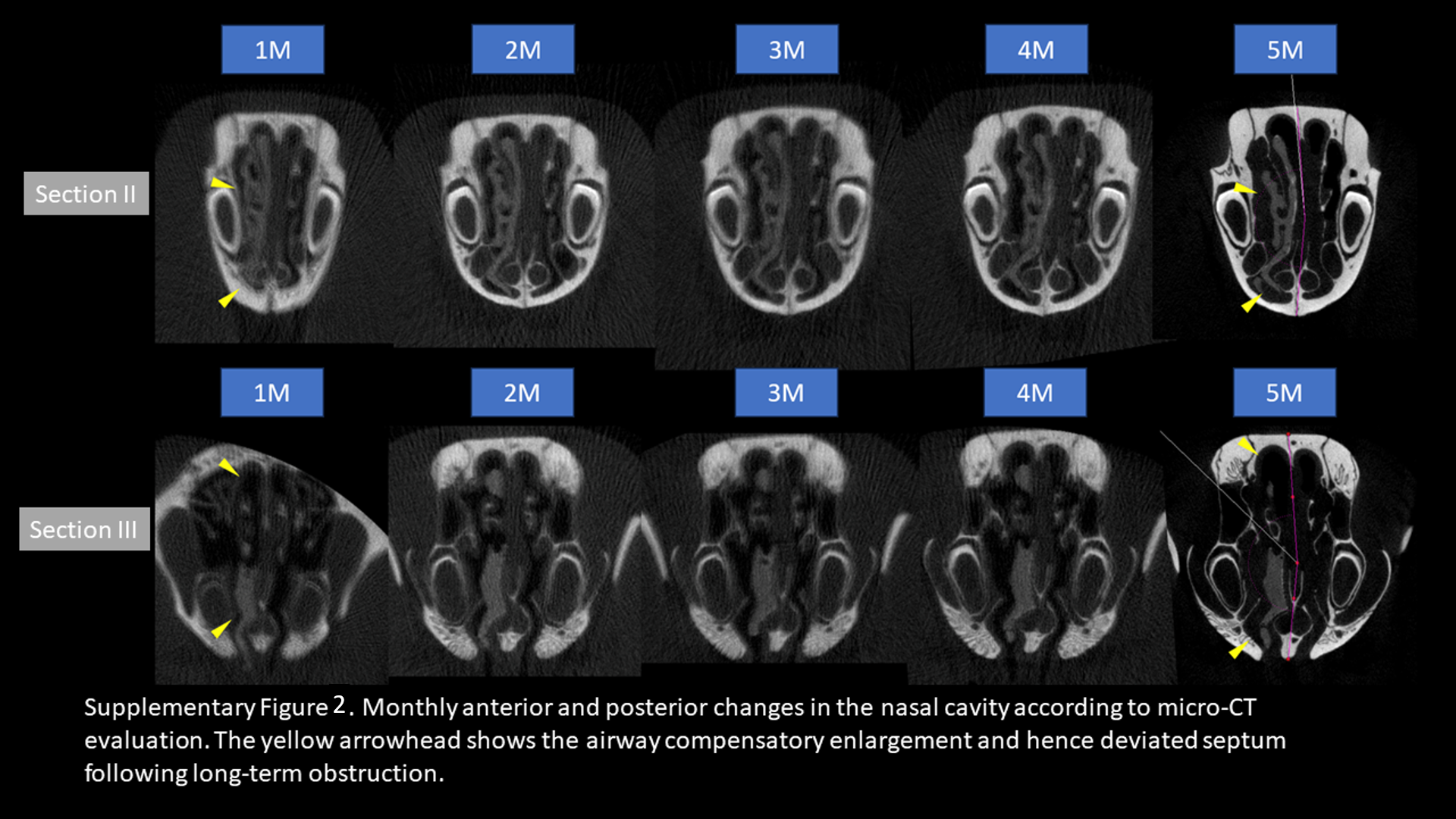

Supplement: Supplementary file 2 — Supplementary Figure 2. [file 41598_2024_51544_MOESM2_ESM.png]
