# Supplementary material for: Craniofacial and olfactory sensory changes after long-term unilateral nasal obstruction—an animal study using MMP-3-LUC transgenic rats
Source: Sci Rep. 2024 Jan 31;14:2616. doi: 10.1038/s41598-024-51544-3 (PMC10830476; doi:10.1038/s41598-024-51544-3)
Supplement: Supplementary file 3 — Supplementary Legends. [file 41598_2024_51544_MOESM3_ESM.docx]

**Supplementary figure legends**

Supplementary figure 1.

*MMP-3*-LUC transgenic rat used in the current study.

Supplementary figure 2.

Monthly anterior and posterior changes in the nasal cavity according to micro-CT evaluation. The yellow arrowhead shows the airway compensatory enlargement and hence deviated septum following long-term obstruction.
